# Nitrogen–phosphorus doped graphitic nano onion-like structures: experimental and theoretical studies†

**DOI:** 10.1039/d0ra10019f

**Published:** 2021-01-13

**Authors:** Armando D. Martínez-Iniesta, Aarón Morelos-Gómez, Emilio Muñoz-Sandoval, Florentino López-Urías

**Affiliations:** División de Materiales Avanzados, IPICYT Camino a la Presa San José 2055, Lomas 4a sección San Luis Potosí 78216 Mexico flo@ipicyt.edu.mx; Global Aqua Innovation Center and Research Initiative for Supra-Materials, Shinshu University 4-17-1 Wakasato Nagano 380-8553 Japan

## Abstract

Onion-like graphitic structures are of great importance in different fields. Pentagons, heptagons, and octagons are essential features of onion-like graphitic structures that could generate important properties for diverse applications such as anodes in Li metal batteries or the oxygen reduction reaction. These carbon nanomaterials are fullerenes organized in a nested fashion. In this work, we produced graphitic nano onion-like structures containing phosphorus and nitrogen (NP-GNOs), using the aerosol assisted chemical vapor deposition method. The NP-GNOs were grown at high temperature (1020 °C) using ferrocene, trioctylphosphine oxide, benzylamine, and tetrahydrofuran precursors. The morphology, structure, composition, and surface chemistry of NP-GNOs were characterized using different techniques. The NP-GNOs showed diameters of 110–780 nm with Fe-based nanoparticles inside. Thermogravimetric analysis showed that NP-GNOs are thermally stable with an oxidation temperature of 724 °C. The surface chemistry analysis by FTIR and XPS revealed phosphorus–nitrogen codoping, and several functionalities containing C–H, N–H, P–H, P–O, P=O, C=O, and C–O bonds. We show density functional theory calculations of phosphorus–nitrogen doping and functionalized C_240_ fullerenes. We present the optimized structures, electronic density of states, HOMO, and LUMO wave functions for P-doped and OH-functionalized fullerenes. The P=O and P–O bonds attributed to phosphates or hydroxyl groups attached to phosphorus atoms doping the NP-GNOs could be useful in improving supercapacitor function.

## Abstract 2

Nitrogen–phosphorus doped graphitic nano onion-like structures.

## Graphical abstract

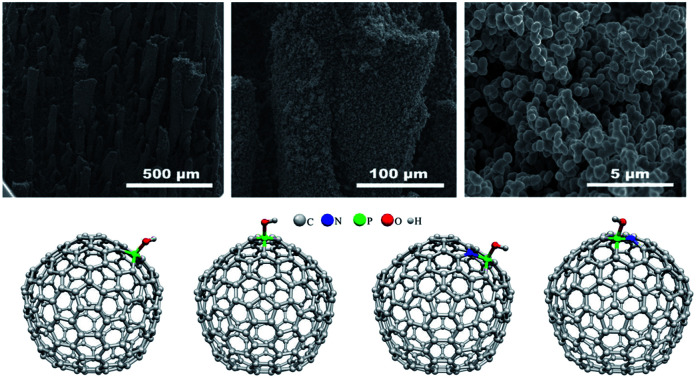

## 1. Introduction

Graphitic nano onion-like particles (GNOs) have attracted attention due to their high surface area, low density, high-temperature stability, and excellent electrical conductivity.^1^ GNOs have been used in lithium batteries,^2^ supercapacitors,^3^ drug delivery,^4^ lubricants,^5,6^ catalyst supports,^7,8^ and removal of contaminants from water.^9^ GNOs have been synthesized using different routes such as arc-discharge,^10^ laser ablation,^11^ and chemical vapor deposition.^12^ In these methods, CH_4_,^13^ toluene,^14^ 1,2-dichlorobenzene,^15^ and tetrahydrofuran (THF)^16^ have been used as precursors. Zhang *et al.*^17^ synthesized GNOs using THF under a reductive hydrogen atmosphere and in the absence of a catalyst. They reported GNOs with diameters ranging from 200 to 600 nm. Graphitic materials have been doped with nitrogen,^18^ boron,^19^ sulfur,^20,21^ beryllium,^22^ and phosphorus.^23,24^ The doping in graphitic materials can improve their electrochemical activity taking advantage of the different electronegativity of the dopants, which can polarize the adjacent atoms.^25^ Recently, extensive investigations on the facile *in situ* synthesis of heteroatom doped carbon nanomaterials for effective catalytic applications have been reported.^26–28^ Chen *et al.*^29^ reported the synthesis of carbon spheres codoped with N and P. They used aniline monomers and phytic acid as nitrogen and phosphorus sources, respectively; their resulting material showed excellent electrocatalytic ORR and OER properties. They also reported using copoded carbon spheres as an electrocatalyst to develop a rechargeable Zn–air battery showing excellent battery performance and cycling stability. Qiao *et al.*^30^ produced carbon nanospheres using an arc-discharge process doped with nitrogen, phosphorus, and iron. They demonstrated that the carbon nanospheres are an efficient electrocatalyst material.

Fullerenes are curved graphene sheets due to the present pentagonal rings.^31–33^ Onion-like structures are large fullerenes arranged in a nested way. Graphitic onions have been produced by high electron irradiation of amorphous carbon or polyhedral particles,^34^ CVD method.^35^ Samanta *et al.*^36^ reported the synthesis of GNOs by pyrolyzing a solution made of nitrogen, hydrolyzed collagen, cobalt, and iron metal as precursors. The obtained material showed the promotion of ORR and OER at low overpotential. Besides, their catalytic behavior has demonstrated superior performance for zinc-air batteries than a traditional catalyst. Furthermore, Ha and coworkers^37^ reported functionalized 3D networks made of GNOs that presented high Li-ion storage. In this work, we have produced NP-GNOs using the AACVD method. We characterize the structure, thermal stability, surface chemistry of NP-GNOs. We show DFT calculations to elucidate the structural stability and electronic properties of C_240_ fullerenes doped with P, N, and functionalized with hydroxyl groups.

## 2. Experimental details

All the chemical reagents used in this work were of chemical grade-purity without further purification purchased from Sigma-Aldrich. The NP-GNOs were produced using the AACVD method (Fig. S1†). This method consists of a quartz tube (length: 90 cm; diameter: 2.5 cm) placed inside a tubular furnace (Barnstead Thermolyne Mod. F21135). The quartz tube is connected to a condenser and an acetone trap for residuals. A solution made of ferrocene (2.5%) as a catalyst, benzylamine (47.43%) as a nitrogen source, tetrahydrofuran (47.43%) as a carbon source, trioctylphosphine oxide (TOPO) (2.5%) as a phosphorus source, and thiophene (0.125%) were set in the sprayer. The nebulized precursors formed inside the sprayer were transported to the quartz tube reactor by Ar (Infra, 99.999%) with a carrier flow at 1 L min^−1^. The temperature for synthesis was 1020 °C for one hour. The GNOs were collected from the reaction quartz tube by scraping its inner walls using a stainless-steel rod. The GNOs yield production was of 1.51 g. The NP-GNOs were characterized by Scanning Electron Microscopy SEM (FEI-Helios NanoLab DualBeam 600 Microscopy), High-Resolution Transmission Electron Microscopy HR-TEM (FEI Tecnai F30), Raman spectroscopy (Micro-Raman Renishaw) using a power laser beam of 532 nm (2.33 eV), thermogravimetric analysis TGA (STA 6000 PerkinElmer) and X-ray diffraction XRD (SmartLab X-ray Diffractometer, Rigaku, Co.) using a power source of Cu (Kα = 1.54 Å).

Electronic calculations were performed using density functional theory (DFT).^38,39^ The generalized gradient approximation with the Perdew, Burke, and Ernzerhof parametrization was chosen for the exchange-correlation functional^40^ as implemented in the SIESTA code.^41^ The wave functions for the valence electrons were represented by a linear combination of pseudo-atomic numerical orbitals using a double-*ζ* polarized basis (DZP),^42^ while the core electrons by norm-conserving Troullier–Martins pseudopotentials in the Kleinman–Bylander non-local form.^43,44^ The real-space grid used for charge and potential integration is equivalent to a plane wave cut-off energy of 150 Ry. Calculations were performed in icosahedral C_240_ fullerene with pentagonal and hexagonal rings. The dopants (phosphorus and nitrogen) were strategically placed in a substitutional fashion in pentagons and hexagons. For a co-doped system (nitrogen and phosphorus doping the graphitic structure simultaneously), the dopants were placed together as dimer N–P into pentagonal and hexagonal rings, replacing two carbon atoms. Density matrix and energy tolerances were both taken as 10^−5^ eV. The geometry optimization was performed by conjugate gradient minimization until the maximum force was <0.03 eV Å^−1^.

## 3. Results and discussion

**Fig. 1:**
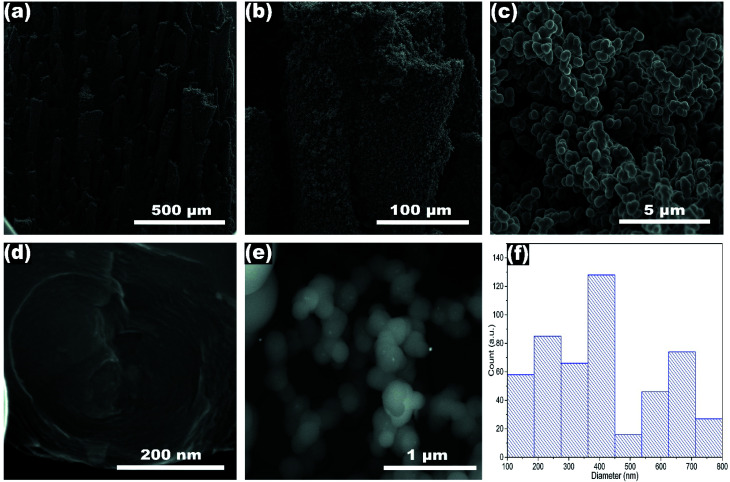
(a and b) SEM images displaying micro towers containing NP-GNOs, (c) a close-up image from a tower made of NP-GNOs, (d) internal structure of a graphitic onion showing a layered material, (e) backscattering SEM image revealing the Fe-nanoparticles inside NP-GNOs, and (f) diameter distribution are showing an average NP-GNOs diameter of 410 nm.

**Fig. 2:**
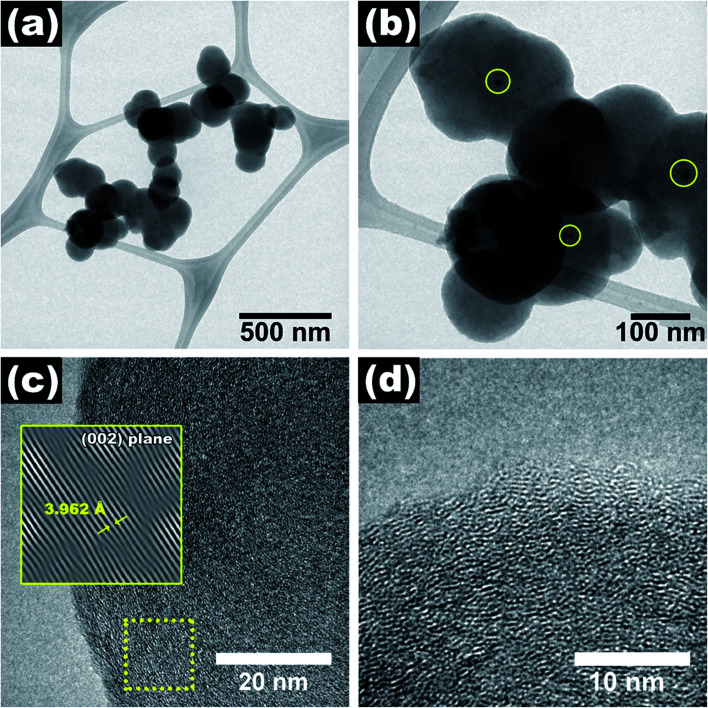
(a and b) TEM and (c and d) HRTEM images of NP-GNOs. In some cases, Fe-based nanoparticles inside the graphite onions were identified.

Fig. 1 shows the SEM images and the average diameter of NP-GNOs. The different magnifications revealing stacked aggregates with hundreds of microns in height, made of spherical-shaped graphitic nanostructures, see Fig. 1a–c. An internal view of a single graphite structure showed the concentric graphitic layers (Fig. 1d). In the backscattering mode, the SEM image revealed that the spherical structures contain a tiny core presumably made of Fe-based material (Fig. 1e) due to ferrocene in the synthesis. We obtained the diameter distribution by counting 500 spherical structures and sampling different sample regions (Fig. 1f), showing an average diameter of 410 nm. Electron diffraction spectroscopy (EDS) characterization revealed that the samples exhibit a high content of carbon (∼92 wt%) and oxygen (∼8 wt%), see Fig. S2.† Although there are several models about the mechanism of growing the carbon onions.^45^ The growth mechanism of these structures is far to be precise (Fig. S3†); however, we believe that because of the high temperature used in the fabrication, our NP-GNOs are building by a gas detonation mechanism.^46^ In this situation, the Fe-based core could be immediately passivated by sulfur, hindering the Fe-based nanoparticle increment. The oxygen could also play a crucial role in Fe-based nanoparticles, favoring the formation of small-size Fe-oxide nanoparticles. It is essential to mention that in a conventional CVD-growth of carbon nanotubes using ferrocene and toluene as precursors (without thiophene and tetrahydrofuran), alpha-Fe and FeC_3_ nanoparticles catalyze the growth of carbon nanotubes. Fig. 2a and b shows the TEM observations, revealing in some cases, a black point centered inside the spherical structure due to the Fe-based nanoparticles; see the yellow circles. The HRTEM images in Fig. 2c and d show an expanded and disordered graphitic structure with an average interlayer distance of 3.962 Å.

**Fig. 3:**
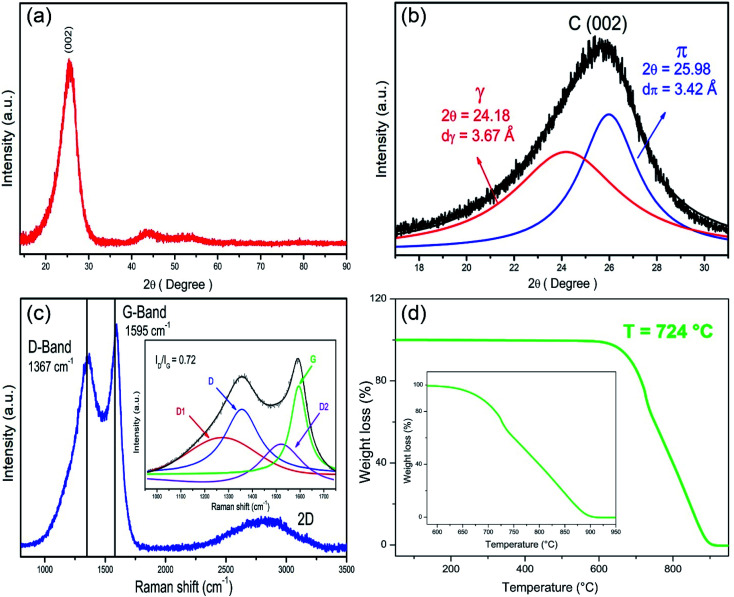
Structural and thermal analysis of NP-GNOs. (a) XRD pattern showing the (002) crystallographic planes related to graphitic materials. (b) Deconvolution over the C (002) signal into two peaks (π and γ). The peak π refers to well-ordered graphitic materials, whereas γ peak indicates the presence of expanded graphitic material. (c) Raman spectrum showing the typical D- and G-peak of graphitic materials. The vertical lines refer to the vibration modes of graphite.^48^ The inset shows the deconvolution analysis of D- and G-band peaks using four Gaussian curves (D1, D, D2, and G). (d) Thermogravimetric analysis showing an oxidation temperature of 724 °C. The inset shows that weight loss began at 600 °C and finished at 915 °C.

Fig. 3a displays the XRD pattern, showing a high-intensity peak at 2*θ* = 25.4° attributed to the (002) crystallographic plane, characteristic of graphitic materials. Furthermore, it is possible to observe low-intensity peaks for 2*θ* around 43° and 55°, which could correspond to Fe-based nanoparticles. Due to its small size, it is difficult for plane indexation. The (002) peak exhibit an asymmetry shape, possibly attributed to expanded graphitic material (EGM). The deconvolution of this peak using pseudovoigt2 curves allows us to identify two more peaks attributed to EGM and well-ordered graphitic material labeled as γ-peak and π-peak, respectively (Fig. 3b). The *d*γ and *d*π interlayer distances were calculated by Bragg's law employing the center of each corresponding peak. The Raman spectrum displays the typical D-band, and G-band centered at 1357 cm^−1^ and 1595 cm^−1^. The ratio of intensities for the D-band and G-band (*I*_D_/*I*_G_) is 0.72, which indicates a graphitic material with defects (Fig. 3c). The deconvolution of these bands revealed the D_1_ band from the graphitic layer edges, and the D_2_ band attributed to the sp^2^–sp^3^ structures probably promoted because of the formation of pentagonal rings.^47^ Fig. S4 and Table S1† shows the deconvoluted Raman spectra for NP-GNOs and pristine-GNOs. We observed that NP-GNOs exhibited a greater contribution of D1 bands than pristine-GNOs. Fig. 3d shows the TGA results displaying an oxidation temperature of 724 °C. The inset shows that the oxidation starts at 600 °C and ends at 915 °C. These results reveal excellent thermal stability, higher than previously reported values for un-doped carbon spheres,^49,50^ indicating that P-doping enhances the thermal stability of GNOs.

**Fig. 4:**
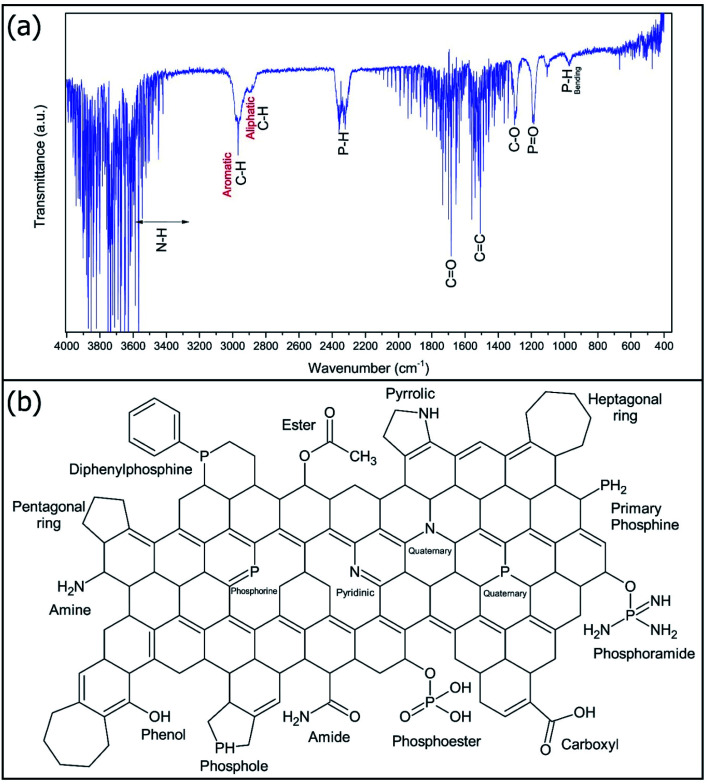
(a) FTIR spectra of NP-GNOs showing the different bonds associated with functionalities containing phosphorus, oxygen, and nitrogen. (b) Schematic representation of oxygen, nitrogen, and phosphorus functionalities in a graphene sheet.

Fig. 4a shows the FTIR spectra of NP-GNOs. Fig. 4b shows the possible chemical species that conform to the NP-GNOs. This analysis reveals the presence of the likely functional groups, which are present in the spheres. Signals at 3500 to 3250 cm^−1^ could be associated with the vibrational modes of N–H corresponding to the amide and amine groups. The valleys at 3000 and 2800 cm^−1^ correspond to aromatic and aliphatic C–H bonds, respectively. The functional groups containing phosphorus are identified at 2330 cm^−1^ as stretching vibrations of P–H bonds. Also, the signals at 970 cm^−1^ can be described as bending motions of P–H bonds associated with primary phosphines and phosphol-like structures, while the vibrations of P=O located in the region of 1200 cm^−1^ can be related whit the presence of phosphoester and phosphoramide groups. The strong signal at 1500 was identified as a C=C sp^2^ bond attributed to graphitic material. Vibrational modes of C=O and C–O are located at 1680 and 1250 cm^−1^, respectively. These vibrational modes could be a consequence of the formation of ester, amide, and carboxyl groups.

**Fig. 5:**
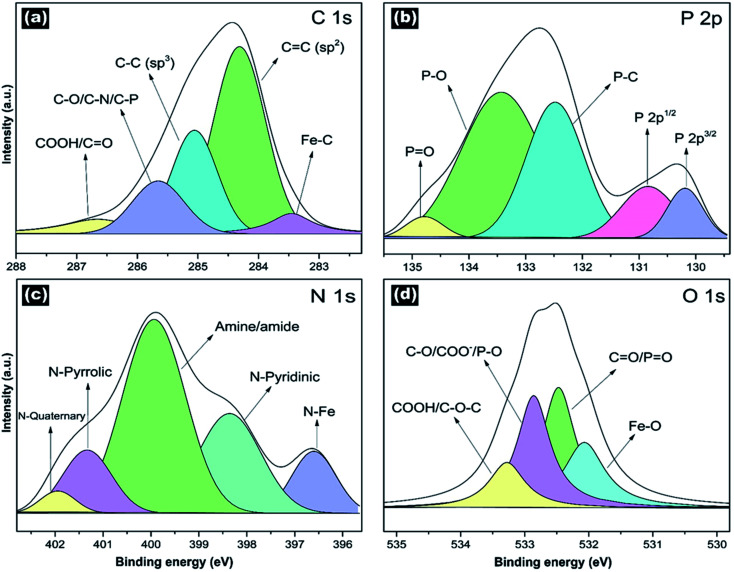
High-resolution XPS spectra of NP-GNOs. Results for (a) C 1s, (b) P 2p, (c) N 1s, and (d) O 1s. The deconvolution analysis shows the different functionalities under each spectrum. The deconvoluted C 1s peak revealed the C=C (sp^2^ hybridization) and C–O (sp^3^ hybridization), Fe–C bonds, C–O/C–N bonds, and the carboxylic groups (COOH). The P 2p spectra revealed the P=O bond and P–O bond attributed to phosphate groups anchored to the GSs surface. The N 1s peak shows different nitrogen functionalities with a remarkable presence of amine and amide functional groups. The O 1s peak shows the presence of hydroxyl groups (C–O), ester groups (COO^−^), carbonyls (C=O), epoxy (C–O–C), and carboxylic groups (COOH).

Fig. 5 shows the high-resolution XPS spectra for C 1s, P 2p, N 1s, and O 1s to study the surface chemistry. Fig. S5† displays the XPS survey. The spectra were deconvoluted to classify the different chemical species. Tables S2–S5† shows results from the deconvolution analysis. The deconvoluted high-resolution C 1s spectra exhibited mainly the sp^2^ and sp^3^ hybridized carbons (Fig. 5a). We identified carboxyl groups (COOH), C–O, C–N, and Fe–C bonds. Fig. 5b displays the deconvoluted high-resolution P 2p spectra, revealing the P–O, P=O, P–C bonds. The P–O and P=O could be attributed to phosphates. The P-doping and P-functionalities provide a high supercapacitor performance (enhanced charge storage and high stability), as was previously reported.^51–53^ The high-resolution deconvoluted N 1s peak (Fig. 5c) showed the different nitrogen doping configurations (N-quaternary, N-pyrrolic, and N-pyridinic) and functionalities (amine, amide, and N–Fe bonds). The O 1s peak confirmed the presence of carboxyl groups. Moreover, we identified the C–O–C, C–O, C=O, and Fe–O bonds (Fig. 5d). C–O could be attributed to either group (methoxy, ethoxy, hydroxyl groups), while C=O could be associated with ester groups.

**Fig. 6:**
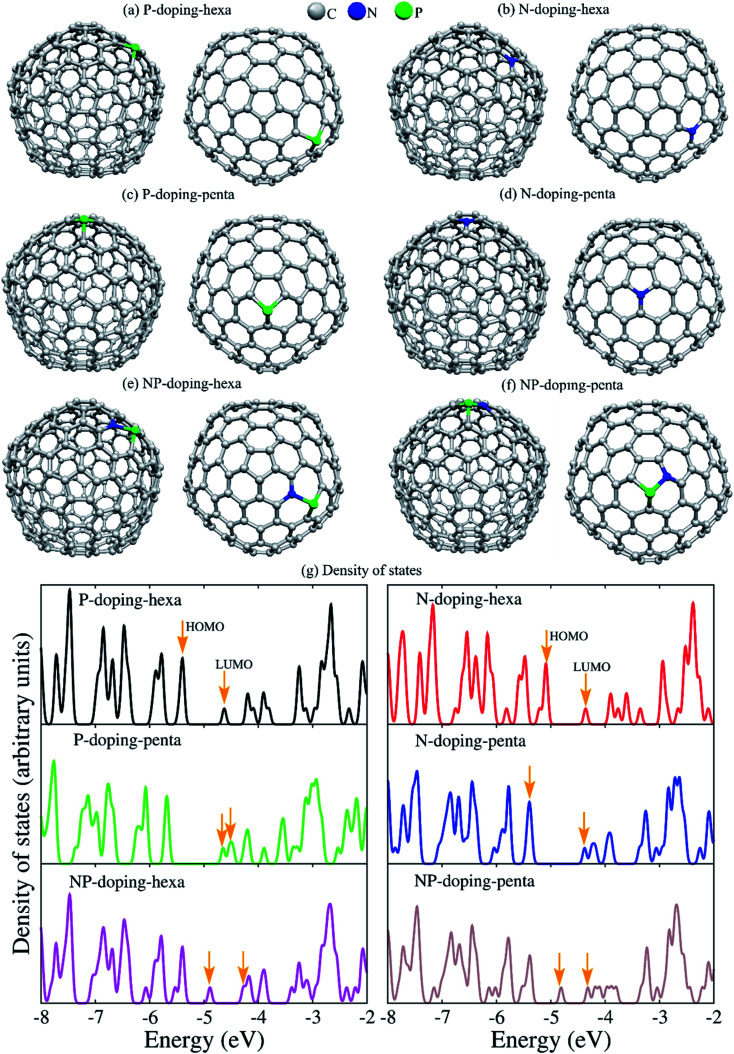
(a–f) Ball-stick model of the optimized doped C_240_ fullerenes (top and side views) showing P-doping, N-doping, and NP-codoping. The dopants were put in hexagonal (hexa) or pentagonal (penta) rings. (g) The corresponding electronic density of states. The arrows indicate the HOMO and LUMO energies.

**Fig. 7:**
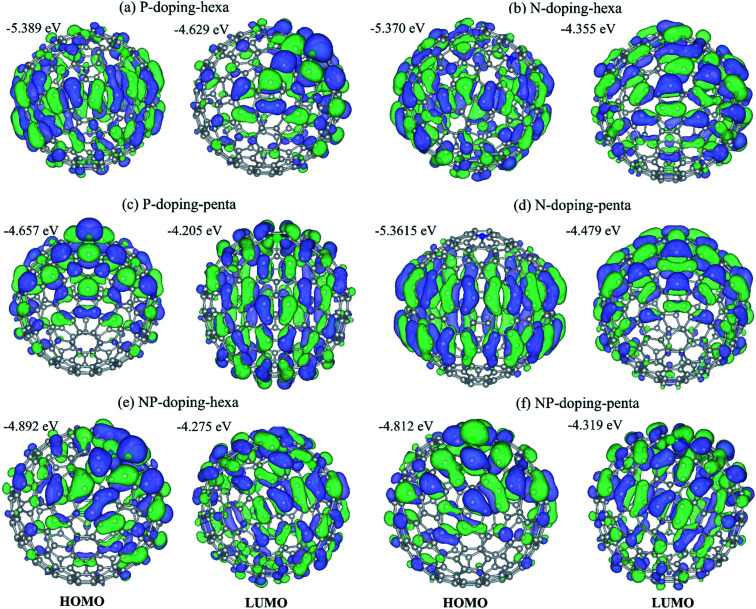
HOMO and LUMO wave functions showing spatial distributions dependent on the dopant and doping configuration. The wave functions were plotted at the isosurface of ±0.03 Å^−3/2^. The corresponding energies are indicated.

To understand the role of phosphorus and nitrogen on the structure and electronic properties of GNOs, we carried out first-principles density functional theory calculations. The theoretical study was performed using the C_240_ fullerene with icosahedral symmetry, and the dopants were placed at pentagonal or hexagonal rings. Fig. 6a–f displays the optimized structures, showing dopants in pentagonal and hexagonal rings. The corresponding density of states (DOS) can be seen in Fig. 6g. The arrows indicate the energy of the highest occupied molecular orbital (HOMO) and the energy of the lowest unoccupied molecular orbital (LUMO). For P-doping-hexa configuration, phosphorus is placed in a hexagonal ring, the interatomic distance between the phosphorus and carbon *d*_PC_ is 1.788–1.823 Å. The phosphorus atom exhibited a Mulliken charge population of 5.385 electrons, indicating a gain of 0.385 electrons. The HOMO wave function is localized on the carbon atoms with a significant presence at the equatorial zone. The LUMO wave function is mainly local at and around the dopant. This system exhibited the lowest LUMO energy, suggesting that P in hexagonal rings could easily be reduced (trap electrons). N-doping-hexa, nitrogen in a hexagonal ring (N-doping-hexa) showed *d*_NC_ of ∼1.42 Å, and the electronic charge is 4.646. The nitrogen injects 0.334 electrons into the fullerene. The HOMO wave function exhibited a delocalization on the carbon atoms with a null presence in the dopant, while the LUMO wave function is localized mainly in the top hemisphere where the dopant is placed. For P-doping-penta, phosphorus in the pentagonal ring showed a *d*_PC_ of 1.799–1.819 Å. The charge gained by P is 0.380 electrons. Contrary to P into hexagonal rings, P in pentagonal exhibited a HOMO wave function a localization at the dopant and around it. This system showed the highest HOMO energy, which means it is energetically less expensive to remove electrons. This fact suggests that P in the pentagonal ring could be easily oxidized. For N-doping-penta, N in a pentagonal ring, the N atoms inject 0.38 electrons to the fullerene structure. The HOMO wave function is localized mainly around the equator, while the LUMO wave function is localized at the top part of the fullerene around the dopant. The NP-doping systems showed reduced bandgap when compared with a single type of dopant. For N and P in a hexagonal ring, P gained 0.324 electrons, while N lost 0.232 electrons. The corresponding HOMO and LUMO wave functions and the associated energies are shown in Fig. 7, showing different spatial distributions dependent on the dopant and doping configuration.

**Fig. 8:**
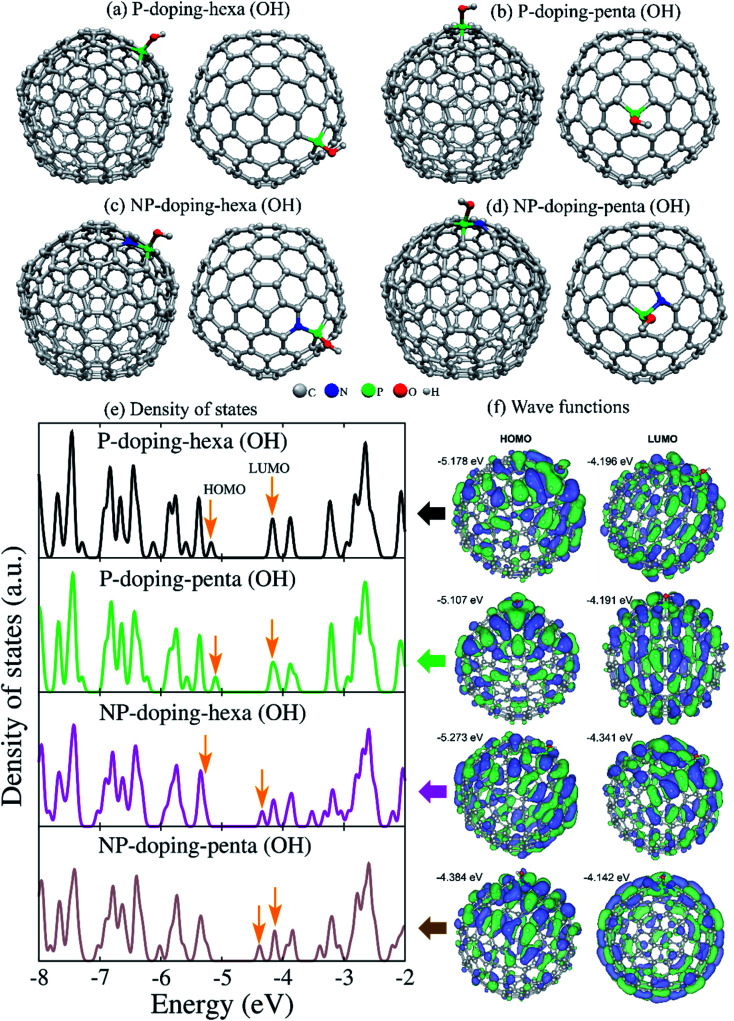
(a–d) Electronic density of states of C_240_ fullerenes doped with phosphorus, nitrogen, and codoped with nitrogen-phosphorus and functionalized with hydroxyl groups (OH). The dopants were put in hexagonal (hexa) or pentagonal (penta) rings. (e) Electronic density of states with the HOMO and LUMO indicated by arrows. (f) HOMO and LUMO wave functions with the corresponding energies showed. The wave functions were plotted at the isosurface of ±0.03 Å^−3/2^.

Fig. 8a–d shows the optimized structures of P- and NP-doped and functionalized C_240_ fullerenes. The N-doped fullerenes are not shown since the OH groups were not covalently joined to N atoms. When the OH group was set on N atoms in NP-doped fullerenes, it migrated to the P atom during the geometry relaxation process. In cyclic voltammetry measurement, the hydroxyl functional groups could be deprotonated in an oxidation process, leaving P=O bonds and returning to the original state (P–OH) in a reduction process. Results for the electronic density of states in Fig. 8e revealed that each structure presents a marked fingerprint. The HOMO and LUMO energies illustrated by the arrows showed a downshift for NP-doping hexa(OH) system, while NP-doping-penta(OH) exhibited reduced bandgap energy. The corresponding HOMO and LUMO wave functions showed different spatial distributions (Fig. 8f). The HOMO wave functions are populated preferentially in the hemisphere containing the functional group.

## 4. Conclusions

In summary, we produced NP-GNOs by the high-temperature synthesis CVD method. The surface's inspection revealed different N-doping configuration (N-pyrrolic, N-quaternary, N-pyridinic) and N-functionalities (amine and amide). The NP-GNOs showed P=O and P–O bonds attributed to phosphates or hydroxyl groups attached to phosphorus atoms doping the graphitic structure. The XPS-survey spectra detected a high oxygen content (∼10%), suggesting oxygen-based functional groups such as amide, carboxyl, carbonyl, phosphates, among others. The NP-GNOs showed approximately 26% of sp^3^ carbon hybridization, indicating the presence of functionalized sites. The NP-GNOs exhibited a high oxidation temperature compared to that reported for carbon nanotubes and carbon spheres. DFT-calculations revealed that phosphorus nitrogen is structurally stable into the graphitic lattice. We found that nitrogen injects electrons, while phosphorus extracts electrons from the carbon structure. The charge injection promoted by nitrogen suffered a reduction when it is incorporated simultaneously with phosphorus. The hydroxyl groups stable and joined covalently joined to phosphorus atoms. The hydroxyl functionalized NP-doping exhibited less profound HOMO energy, which could be easily oxidized. We envisage that our synthesized material could be used as an electrode in lithium-ion batteries and flame-retardant materials.

## Author contributions

EMS. AMG, and FLU designed the research. ADMI conducted the experiments and analyzed the data.

## Conflicts of interest

The authors declare no competing financial interests.

## Supplementary Material

RA-011-D0RA10019F-s001
