# Supplementary material for: Nitrogen–phosphorus doped graphitic nano onion-like structures: experimental and theoretical studies
Source: RSC Adv. 2021 Jan 13;11(5):2793–803. doi: 10.1039/d0ra10019f (PMC8693872; doi:10.1039/d0ra10019f)
Supplement: RA-011-D0RA10019F-s001 [file RA-011-D0RA10019F-s001.pdf]

Electronic Supplementary Material (ESI) for RSC Advances.
This journal is © The Royal Society of Chemistry 2021

Supplementary Information

Nitrogen-Phosphorus Doped Graphitic Nano Onion-Like Structures: Experimental and Theoretical Studies

Armando D. Martínez-Iniesta[1], Aarón Morelos-Gómez[2], Emilio Muñoz-Sandoval[1], Florentino López-Urías[1]*

[1]División de Materiales Avanzados, IPICYT, Camino a la Presa San José 2055, Lomas 4a sección, San Luis Potosí, S.L.P., 78216, México.
[2] Global Aqua Innovation Center and Research Initiative for Supra-Materials, Shinshu University, 4-17-1 Wakasato, Nagano 380-8553, Japan.

Fig. S1: Armando D. Martínez-Iniesta et al.

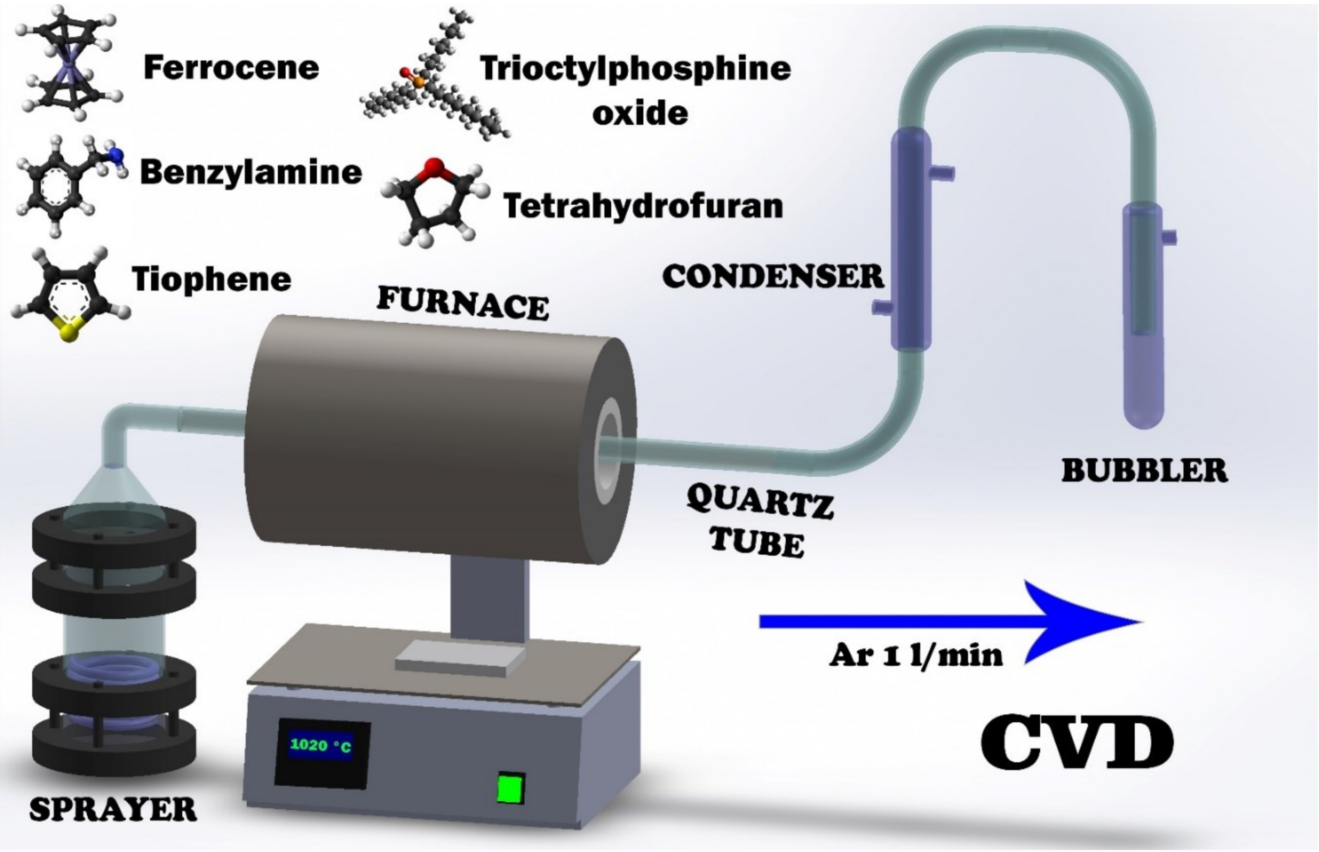

Fig. S1: Schematic representation of aerosol assisted chemical vapor deposition (AACVD) setup used to synthesize nitrogen-phosphorus graphite onion-like structures (NP-GNOs). The NP-GNOs were grown at 1020°C for one hour. The NP-GNOs were collected from the quartz tube by scraping the inner walls. The ball-stick models of the different precursors are shown.

Fig. S2: Armando D. Martínez-Iniesta et al.

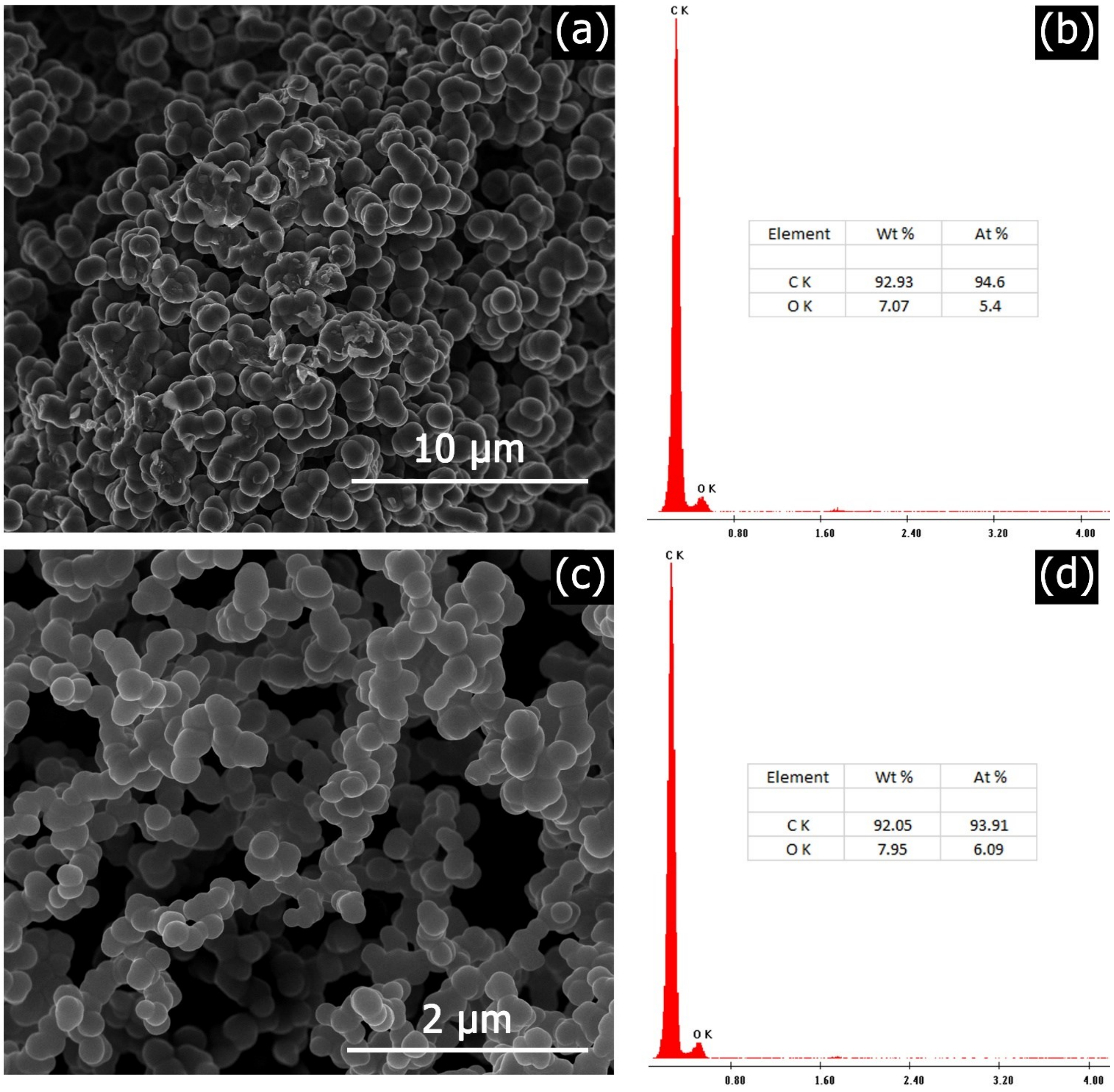

Fig. S2: (a) and **(c)** SEM images of NP-GNOs and their corresponding elemental analysis by EDS **(b)** and **(d)**.

Fig. S3: Armando D. Martínez-Iniesta et al.

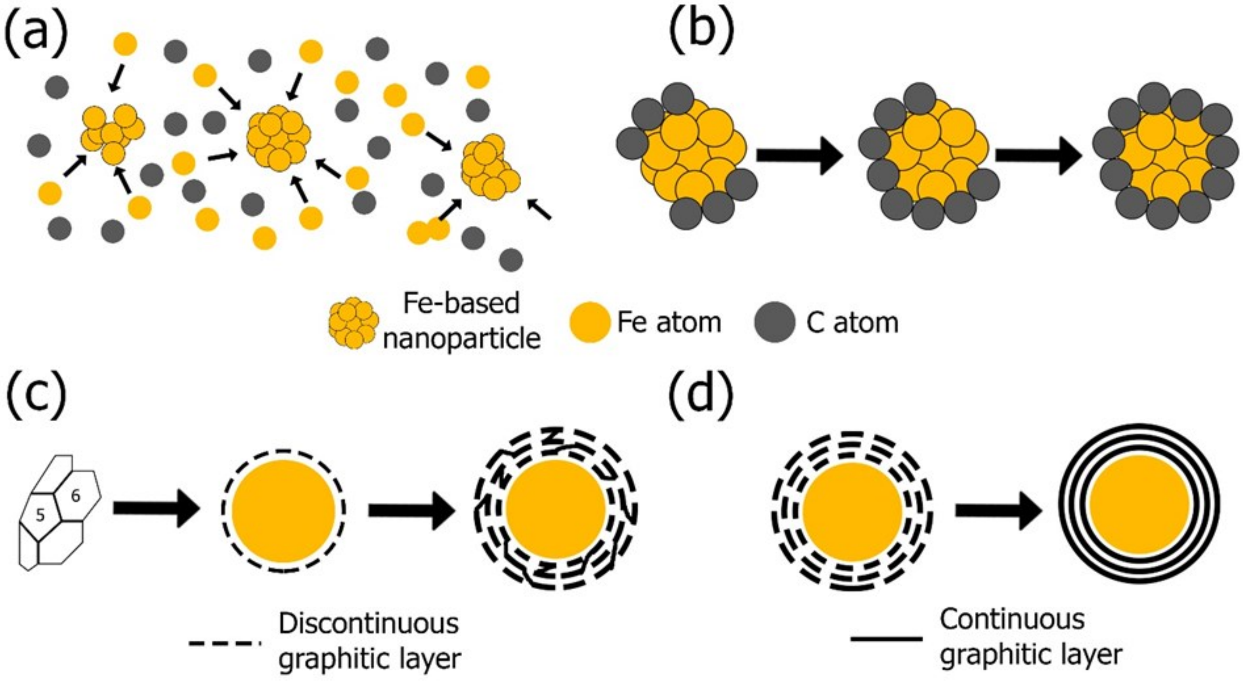

Fig. S3: Possible schematic of NP-GNOs growth mechanism. **(a)** The Fe atoms from ferrocene decomposition are brought together, forming small Fe-clusters. **(a)** Fe-nanoparticles are surrounded by carbon released from ferrocene and other precursors (benzylamine, tetrahydrofuran, thiophene, and trioctylphosphine oxide). In this process, the carbon can be accompanied by nitrogen, phosphorus, and oxygen. **(c-d)** Formation of Fe/graphitic core-shell nanoparticles.

Fig. S4: Armando D. Martínez-Iniesta et al.

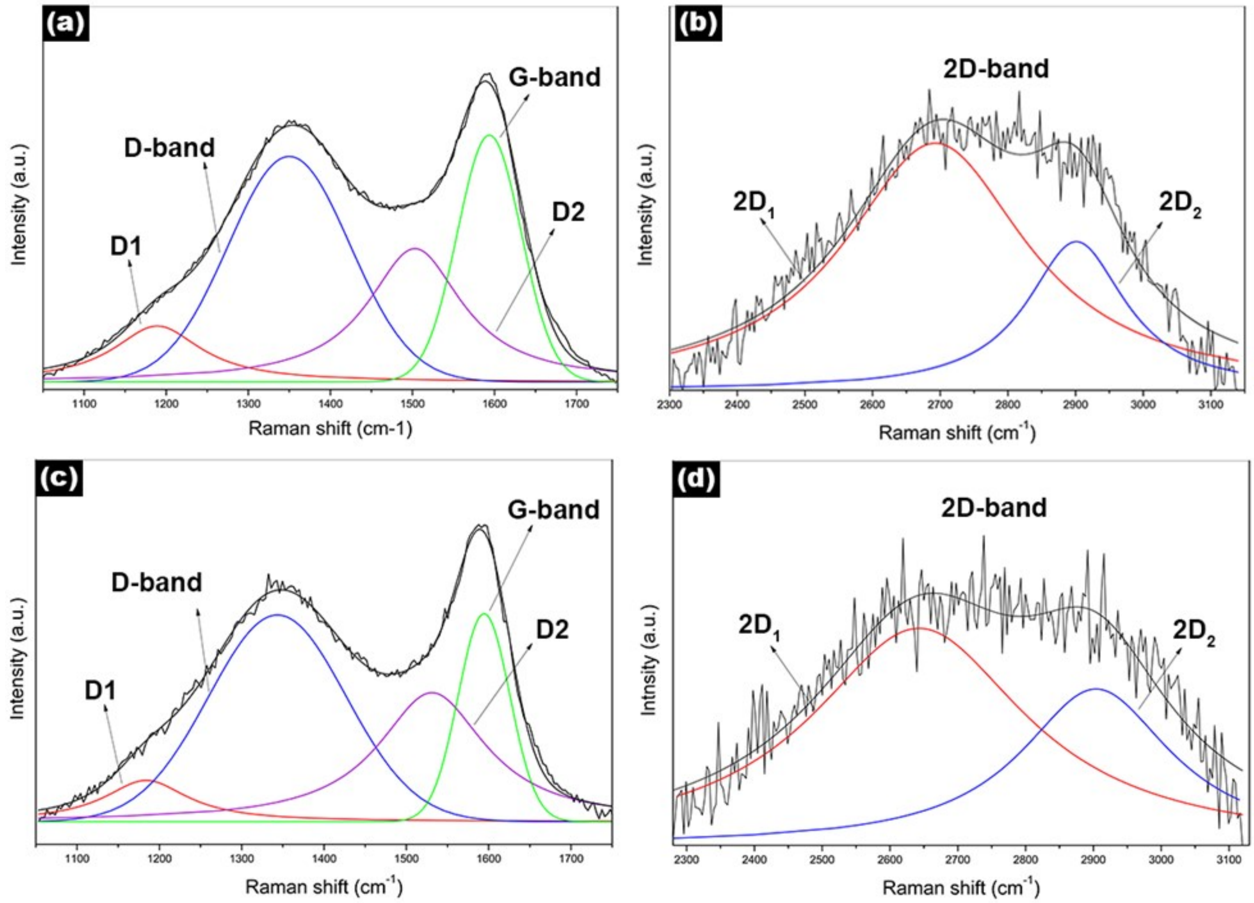

Fig. S4: Raman spectra deconvolution analysis for **(a-b)** NP-GNOs and **(c-d)** pristine-GNOs. The deconvolution of Raman spectra using Lorentz and Gaussian fitting (see Table S1). Notice that there are two more peaks, the formation of D2-peak and D1-peak attributed to the influence in the sp^2 vibration modes by $-sp^3$ C-H bonding, C-O bonding, and amorphous carbon.

Fig. S5: Armando D. Martínez-Iniesta et al.

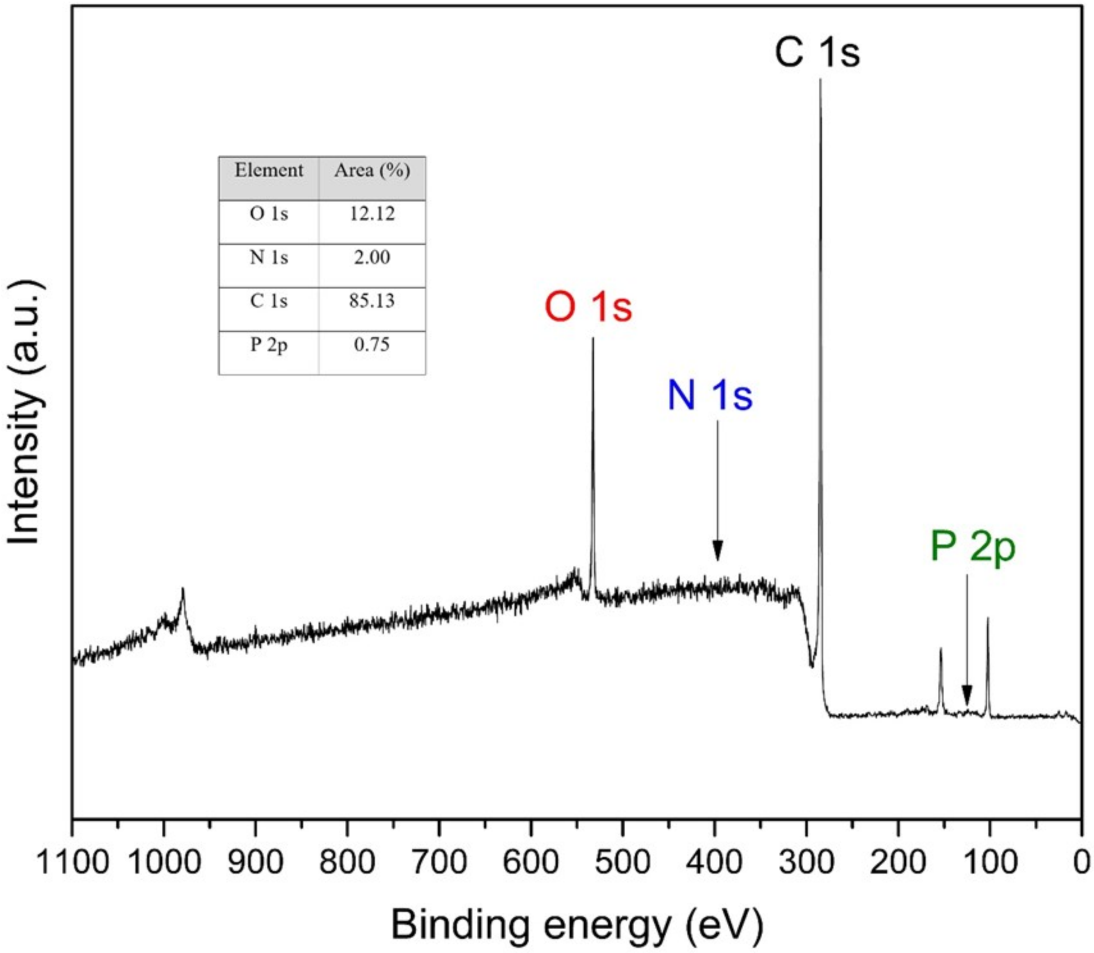

Fig. S5: XPS survey of NP-GNOs showing the involved chemical elements.

Table S1: Armando D. Martínez-Iniesta et al.

Band	Center (eV)	FWHM	Area (%)	Peak type
**NP-GNOs**				
D1	1189.07126	130.56128	9.45569	Lorentz
D	1349.90204	174.91736	41.548	Gaussian
D2	1503.05972	140.05468	25.19844	Lorentz
G	1593.74567	91.54132	23.79786	Gaussian
$2D_1$	2692.61773	329.15924	73.63682	Lorentz
$2D_2$	2900.97444	177.44249	26.36318	Lorentz
**Pristine-GNOs**				
D1	1183.92523	123.82133	7.1006	Lorentz
D	1343.41408	196.60462	46.30472	Gaussian
D2	1530.99408	158.55874	29.06389	Lorentz
G	1594.37783	73.95061	17.53079	Gaussian
$2D_1$	2642.74319	386.75381	65.51345	Lorentz
$2D_2$	2903.7307	270.70193	34.48655	Lorentz

Table S1: Data from Raman peaks deconvolution analysis for pristine (undoped) and NP-GNOs. Results for bands (D, D1, D2, G, 2D1, 2D2), the maximal intensity peaks position (center), full width at half maximum (FWHM), and the area under each curve, and curve-type used in the fitting.

Table S2: Armando D. Martínez-Iniesta et al.

Chem. specie	Center (eV)	FWHM	Area (%)	Peak type
**C 1s**				
COOH/C=O	286.65517	1.40102	6.34504	Lorentz
C-O/C-N/C-P	285.65552	1.00787	14.01249	Gaussian
C-C (sp^3)	285.05737	0.8852	24.14133	Gaussian
C=C (sp^2)	284.31446	1.01163	49.89124	Gaussian
Fe-C	283.45696	0.81379	5.60986	Lorentz

Table S2: Deconvolution data for C1s peak.

Table S3: Armando D. Martínez-Iniesta et al.

Chem. specie	Center (eV)	FWHM	Area (%)	Peak type
**P 2p**				
P=O	134.78238	0.77734	3.25223	Gaussian
P-O	133.42952	1.66479	46.94409	Gaussian
P-C	132.48108	1.1981	31.50545	Gaussian
P $2p^{1/2}$	130.84083	1.11779	11.20512	Gaussian
P $2p^{3/2}$	130.18969	0.73963	7.09312	Gaussian

Table S3: Deconvolution data for P2p peak.

Table S4: Armando D. Martínez-Iniesta et al.

Chem. specie	Center (eV)	FWHM	Area (%)	Peak type
**N 1s**				
N-Quaternary	401.94385	0.86928	3.13289	Gaussian
N-Pyrrolic	401.33066	1.20409	12.34172	Gaussian
Amine/amide	399.93583	1.55315	48.90862	Gaussian
N-Pyridinic	398.36004	1.53916	24.93183	Gaussian
N-Fe	396.60028	1.08285	10.68494	Gaussian

Table S4: Deconvolution data for N1s peak.

Table S5: Armando D. Martínez-Iniesta et al.

Chem. specie	Center (eV)	FWHM	Area (%)	Peak type
**O 1s**				
COOH-C-O-C	533.27781	0.68103	13.52241	Lorentz
C-O/COO^-/P-O	532.85987	0.62359	30.80462	Lorentz
C=O/P=O	532.47211	0.63717	33.74019	Lorentz
Fe-O	532.06989	0.77418	21.93278	Lorentz

Table S5. Deconvolution data for O1s peak.
